# The perioperative brain–heart–immune axis: autonomic resilience, multimodal monitoring and postoperative delirium

**DOI:** 10.3389/fmed.2026.1906375

**Published:** 2026-08-14

**Authors:** Eva Maria Aldana, Cristian Aragón-Benedí, César Aldecoa

**Affiliations:** 1 Hospital Vithas Xanit Internacional, Benalmádena, Spain; 2 Neuroscience Section, Spanish Society of Anesthesiology, Resuscitation, and Pain Therapeutics SEDAR, Madrid, Spain; 3 Hospital Universitario Miguel Servet, Zaragoza, Spain; 4 Instituto de Investigacion Sanitaria Aragon, Zaragoza, Spain; 5 Universidad de Zaragoza Facultad de Medicina, Zaragoza, Spain; 6 Hospital Universitario Río Hortega, Valladolid, Spain; 7 Universidad de Valladolid Facultad de Medicina, Valladolid, Spain; 8 Instituto de Investigación Biosanitaria de Valladolid (IBioVALL), Valladolid, Spain

**Keywords:** autonomic nervous system, baroreflex sensitivity, frailty, heart rate variability, multimodal monitoring, postoperative delirium, prehabilitation

## Abstract

Postoperative delirium (POD) is the most frequent acute neurological complication in the older surgical patient and is independently associated with increased mortality, accelerated cognitive decline and loss of functional independence. Models centred on isolated organs do not explain why systemic frailty predicts perioperative cognitive failure irrespective of the type of surgery or the anaesthetic technique. We have conducted a hypothesis-generating narrative review that synthesises the evidence positioning the autonomic nervous system (ANS) as the link between frailty, the surgical stress response and POD, and that articulates a perioperative conceptual framework—the brain–heart–immune axis—in which the vagus nerve integrates an autonomic-cardiovascular arm, a neuroimmune arm mediated by the cholinergic anti-inflammatory pathway and a neurocognitive arm. The most recent studies suggest that indices of autonomic adaptability —rather than markers of tone or state—may independently predict POD and may modulate the postoperative neuroinflammatory trajectory; that preoperative baroreflex sensitivity may anticipate post-induction hypotension; that certain electroencephalographic recordings may be associated with greater POD risk; and that non-invasive vagal neuromodulation might reduce its incidence. On this basis, a four-phase perioperative strategy is proposed—autonomic assessment, multimodal prehabilitation, multimodal intraoperative monitoring and postoperative protection. Its status should be specified: the brain–heart–immune axis is proposed as an integrative, hypothesis-generating conceptual framework, not as a validated clinical model, since the evidence linking heart rate variability (HRV) and baroreflex sensitivity (BRS) with POD is predominantly observational, heterogeneous and of limited certainty; for this reason, neither HRV nor BRS should yet be employed in isolation as diagnostic or predictive tools, and the framework requires prospective validation and methodological standardisation of the autonomic indices before its incorporation into risk stratification and delirium prevention.

## 1 Introduction

Postoperative delirium (POD) constitutes one of the perioperative complications of greatest clinical and socioeconomic impact in the older surgical patient. The available epidemiological evidence indicates that its incidence lies between 20 and 40% in individuals older than 60 years undergoing major surgery (1–3), with broader ranges of 13 to 41% in unselected surgical populations that reflect the heterogeneity of procedures and clinical profiles. In contexts of greater physiological vulnerability—cardiac surgery, admission to intensive care units—the incidence may exceed 55%, evidencing a clear risk gradient associated with the surgical burden and the patient’s baseline state (4, 5). Beyond its prevalence, POD is independently associated with an increase in 30-day, 90-day and one-year mortality, acceleration of cognitive decline towards chronic neurocognitive disorder, longer hospital stay and institutionalisation, as well as deterioration of quality of life and long-term functional independence. A recent editorial holds that POD remains an unresolved challenge, with a persistent gap between the available international evidence and its translation into routine perioperative practice (6).

The aetiopathogenic models of POD have evolved from single-cause approaches—predominantly centred on the cholinergic hypothesis or on the systemic inflammatory response—towards progressively multifactorial conceptualisations that recognise the interaction between prior neural vulnerability and perioperative triggers (1, 5, 7–11). Models centred on isolated organs present intrinsic limitations in explaining the consistency with which systemic frailty predicts POD independently of the type of surgery, the anaesthetic technique or the origin of the inflammatory response, because the perioperative response to surgical trauma is not organised by isolated organs, but is coordinated through complex neurophysiological, cardiovascular, inflammatory and metabolic networks.

In this context, the brain–heart–immune axis (BHI-A) emerges as an integrative conceptual framework (12). Advanced age, frailty, systemic inflammation, acute pain, hypovolaemia, sleep disturbance and anaesthetic exposure converge upon autonomic regulation (13–15). These factors may produce sympathetic predominance, reduced parasympathetic modulation, altered baroreflex function and diminished cardiovascular adaptability. In this context, autonomic dysfunction is not simply a cardiovascular phenomenon, but a marker of reduced systemic resilience.

The autonomic nervous system (ANS) occupies a potentially important position within the brain–heart–immune axis by integrating signals related to physiological reserve, haemodynamic regulation, and inflammatory control through pathways that include the cholinergic anti-inflammatory pathway. Autonomic dysfunction may therefore represent a candidate convergence node through which impaired cardiovascular adaptability, altered inflammatory responses, neurotransmitter disturbances, cerebral hypoperfusion, and disrupted neural-network function interact to increase susceptibility to postoperative delirium (POD). However, current evidence does not establish that autonomic dysfunction determines, mediates, or constitutes a direct causal mechanism of POD. Observed autonomic alterations may instead represent a pre-existing marker of reduced physiological reserve, a consequence of surgical stress or evolving cerebral dysfunction, a correlate of shared causes such as ageing, frailty, comorbidity, medication exposure, inflammation, or impaired sleep, or some combination of these mechanisms. Within this framework, heart rate variability (HRV) provides a non-invasive measure of cardiac autonomic dynamics and has been associated with perioperative vulnerability, but it should not be interpreted as a direct measurement of central autonomic activity, cholinergic anti-inflammatory pathway function, or global physiological reserve (16). Accordingly, associations between preoperative HRV-derived indices, inflammatory trajectories, haemodynamic instability, and POD are compatible with the proposed brain–heart–immune framework and support further mechanistic investigation, but they do not confirm or validate a specific causal sequence.

The perioperative period should not be conceived as a source of risk, but as an exceptional window of clinical opportunity: the moment at which autonomic dysfunction can be quantified, stratified and intervened upon, before the pathophysiological cascade of delirium is triggered. Non-invasive measures of autonomic function, such as heart rate variability (HRV) and baroreflex sensitivity (BRS), may help to estimate the patient’s physiological reserve and their vulnerability to perioperative stress.

This review aims to synthesise the available evidence on the mechanisms through which the ANS acts as the link between frailty, the surgical stress response and POD, and to propose an integrative model with diagnostic, monitoring and therapeutic implications for the perioperative context.

In this context, we propose the hypothesis that preoperative autonomic resilience, assessed non-invasively by means of heart rate variability and baroreflex sensitivity as an expression of the organism’s capacity to adapt to surgical stress, might represent the physiological link between preoperative frailty and the mechanisms implicated in the development of postoperative delirium. From this perspective, its assessment might provide a novel approach to identifying the vulnerable brain before surgery and, if confirmed, improve risk stratification and guide personalised prehabilitation strategies.

## 2 Methodology of the narrative review and scope

### 2.1 Sources of information and search strategy

This hypothesis-generating narrative review drew upon a structured and reproducible bibliographic search in PubMed/MEDLINE, Scopus, Web of Science and Google Scholar, confined to the period between 1 January 2016 and 1 March 2026, complemented by manual scrutiny of the reference lists of the included articles (backward citation tracking) to retrieve additional relevant studies and supplemented by earlier seminal publications. The strategies combined the terms “postoperative delirium,” “autonomic nervous system,” “heart rate variability,” “baroreflex sensitivity,” “frailty,” “brain–heart axis,” “brain–heart–immune axis,” “neuroinflammation,” “blood–brain barrier,” “electroencephalography,” “nociception monitoring,” “vagus nerve stimulation” and “prehabilitation.”

Priority was given to recent clinical guidelines, systematic reviews, meta-analyses, prospective observational studies and randomised controlled trials directly relevant to perioperative autonomic dysfunction and postoperative neurocognitive outcomes (13, 14, 16–18). As this is a narrative synthesis, no systematic methodology (PRISMA/PROSPERO) was applied nor was any meta-analysis performed: the purpose was not to obtain pooled effect estimates, but to integrate the available evidence within a framework that links autonomic resilience, the perioperative stress response and postoperative delirium.

The initial search retrieved 1,449 records; after screening of titles and abstracts, 187 were selected for full-text reading, of which 65 were incorporated into the synthesis, together with 9 additional references derived from the manual scrutiny. Discrepancies between the authors were resolved by discussion and consensus.

The eligibility criteria were:

Inclusion criteria: (i) studies in adult humans (≥18 years) or mechanistic and translational studies directly relevant to perioperative autonomic physiology; (ii) peer-reviewed publications in English or Spanish; (iii) designs encompassing randomised controlled trials, meta-analyses, systematic reviews, prospective or retrospective observational studies, mechanistic studies, consensus documents or clinical practice guidelines; and (iv) outcomes related to HRV, BRS, EEG, nociception monitoring, vagal neuromodulation, frailty, neuroinflammation or perioperative neurocognitive outcomes. Exclusion criteria: conference abstracts without full text, letters without original data, delirium that was exclusively non-surgical or paediatric, and studies whose methodological quality or relevance was judged insufficient after full-text reading.

Study selection and data extraction were performed independently by the authors, with discrepancies resolved by discussion and consensus. For each study, the design, the population, the sample size, the autonomic index or the intervention evaluated, the neurocognitive or haemodynamic outcome and the principal limitations were recorded.

As this is a narrative review, no formal risk-of-bias assessment nor the PRISMA framework was applied; instead, the quality and transparency of the process were structured in accordance with the SANRA recommendations (Scale for the Assessment of Narrative Review Articles) (19), and the robustness of each source was graded according to the levels of evidence of the Oxford Centre for Evidence-Based Medicine (20). This classification explicitly distinguishes randomised controlled trials and meta-analyses (levels 1–2), prospective and retrospective observational studies (levels 2–3), and mechanistic studies, narrative reviews, consensus documents and expert opinion (levels 4–5). The OCEBM level is incorporated into the summary tables (Tables 1–6) so that the reader may weigh the strength of the empirical substrate supporting each assertion and avoid attributing to the model an empirical support greater than that actually available.

**Table 1:** The individual heart-rate-variability and baroreflex-sensitivity indices.

Index	Physiological correlate	Perioperative evidence	Principal limitations	References
SDNN	Global HRV; overall autonomic modulation	Most consistent HRV index associated with delirium in meta-analysis; total power <650 ms^2^ → post-induction hypotension	Sensitive to recording duration, artefacts, arrhythmias	(13, 16, 35)
RMSSD	Short-term, predominantly parasympathetic modulation	↓ in frailty; weak or inconsistent association with POD	Influenced by respiration, extrasystoles, medication	(16, 32)
HF power	Respiratory-band, predominantly parasympathetic modulation	Elevated preoperative HF peak associated with ↑ POD (OR 1.89)	Affected by ventilation and respiratory pattern	(36, 40)
LF power	Mixed sympathetic, parasympathetic and baroreflex influences	Provides autonomic-regulatory information; complex interpretation	Should not be read as a pure sympathetic marker	(36)
LF/HF	Ratio historically termed “sympatho-vagal balance”	No reliable standalone value	Physiologically oversimplified; limited independent value	(13, 36)
DFAα1	Short-term detrended-fluctuation-analysis scaling exponent (short-range R–R correlations)	Independent predictor of POD (OR 0.47; p = 0.007); <1.05 → postoperative AF; associated with an attenuated IL-6 trajectory	Methodologically demanding; context-dependent	(40) (HiPPIE); (13)
SD2/SD1	Poincaré long−/short-term variability ratio	Independent predictor of POD (OR 0.44; p = 0.008); ↑ values → ↓ IL-6 (β = −0.22; *p* = 0.009)	Advanced processing; non-standardised thresholds	(40) (HiPPIE)
BRS	Baroreceptor–vagal (arterial–cardiac) arc gain	<9.26 ms/mmHg → post-induction hypotension (AUC 0.747; Se 57.7%, Sp 84.4%); −35% intraoperatively in neurosurgery	Influenced by age, vascular stiffness, anaesthetics, method	(21, 25, 39)
ANI/ANIm	Processed HRV proxy (SDNN + HFnu; ANI-monitor algorithm)	Validated for intraoperative nociception; preoperative frailty/Prehab use described only in a protocol (PreANI)	Preoperative frailty/reserve use not yet validated	(35, 47)

OCEBM (20) evidence levels for each Brain–Heart–Immune Axis component are reported in Table 4; the proposed state-versus-adaptability classification is presented separately in Table 2. AF, atrial fibrillation; ANI/ANIm, Analgesia Nociception Index / ANI monitor; ANS, autonomic nervous system; AUC, area under the curve; BRS, baroreflex sensitivity; DFAα1, short-term detrended fluctuation analysis scaling exponent; HF, high-frequency power; HFnu, high-frequency power in normalised units; HiPPIE, (40) prospective study; HRV, heart rate variability; IL-6, interleukin-6; LF, low-frequency power; OR, odds ratio; POD, postoperative delirium; Prehab, prehabilitation; RMSSD, root mean square of successive differences; SD1/SD2, Poincaré-plot short−/long-term descriptors; SDNN, standard deviation of NN intervals; Se, sensitivity; Sp, specificity.

**Table 2:** State-versus-adaptability framework.

Index	Proposed classification	Empirical basis for the assignment	Degree of uncertainty
State markers — reflection of the current operating level of the ANS (snapshot)			
SDNN	State	Global HRV; most consistent state–POD association in meta-analysis	Moderate: association only; direction inconsistent across settings
RMSSD	State	Rapid parasympathetic modulation; weak POD association	High: inconsistent and heavily confounded
HF power	State	Predominantly parasympathetic; paradoxical ↑ HF-peak with ↑ POD illustrates that a high state ≠ adaptability	High: single cohort; paradoxical direction
LF power	State	Mixed autonomic regulation; regulatory information only	High: non-specific
LF/HF	State (use with caution)	Included only to signal that it is not a valid stand-alone index of sympatho-vagal balance	Very high: not recommended as a classifier
Adaptability markers — dynamic capacity for autonomic modulation (functional reserve)			
DFAα1	Adaptability	Independent POD predictor in one prospective cohort (HiPPIE); convergent signal with postoperative AF (13)	High: single-centre, exploratory; thresholds not standardised
SD2/SD1	Adaptability	Independent POD predictor in the same cohort; proposed — not validated — as an index of autonomic flexibility/reserve	High: single-centre, exploratory
Composite/proxy indices			
BRS	State + reserve	Predicts post-induction hypotension; no direct POD endpoint	Moderate–high: surrogate (haemodynamic) endpoint
ANI/ANIm	State proxy	Validated for nociception; state/reserve framing only within a protocol (PreANI)	High: reserve framing unvalidated

The classification of indices as state versus adaptability markers is an interpretive and currently unvalidated conceptual framework proposed by the authors; it is presented to structure discussion, not as settled physiological terminology, and requires prospective validation. The degree of uncertainty reflects the authors’ qualitative appraisal of the current supporting evidence and is intentionally conservative. AF, atrial fibrillation; ANI/ANIm, Analgesia Nociception Index/ANI monitor; ANS, autonomic nervous system; BRS, baroreflex sensitivity; DFAα1, short-term detrended fluctuation analysis scaling exponent; HF, high-frequency power; HiPPIE, (40) prospective study; IL-6, interleukin-6; LF, low-frequency power; POD, postoperative delirium; PreANI, PreANI study protocol; SD1/SD2, Poincaré-plot descriptors; SDNN, standard deviation of NN intervals.

**Table 3:** Signal-acquisition and preprocessing considerations for the heart-rate-variability and baroreflex-sensitivity indices.

Index	Signal-acquisition and preprocessing considerations
SDNN	Sensitive to recording duration, motion artefacts and arrhythmias.
RMSSD	Influenced by respiration, ectopic beats and medication.
HF power	Affected by ventilation and respiratory pattern.
LF power	Mixed physiological origin; should not be read as a pure sympathetic marker.
LF/HF	Oversimplified ratio; limited independent value.
DFAα1	Methodologically demanding; context-dependent computation of the short-term scaling exponent.
SD2/SD1	Advanced non-linear processing; thresholds not standardised.
BRS	Influenced by age, vascular stiffness, anaesthetics and measurement method; acquisition method not standardised.
ANI/ANIm	Proprietary processing of SDNN and normalised HF; preoperative use not standardised.

Table summarising the principal signal-acquisition and preprocessing considerations for each index, consolidated from the limitations in Table 1. ANI/ANIm, Analgesia Nociception Index/ANI monitor; BRS, baroreflex sensitivity; DFAα1, short-term detrended fluctuation analysis scaling exponent; HF, high-frequency power; HFnu, high-frequency power in normalised units; LF, low-frequency power; RMSSD, root mean square of successive differences; SD1/SD2, Poincaré-plot descriptors; SDNN, standard deviation of NN intervals.

**Table 4:** Synthesis of the evidence by component of the brain–heart–immune axis.

Axis component	Principal evidence (studies)	Predominant design	OCEBM level	Current certainty	Clinical implications	Research priorities
Autonomic arm (HRV: DFAα1, SD2/SD1)	DFAα1 and SD2/SD1 predict POD; paradoxical HF association (16, 40)	Prosp. cohort; SR+MA	1–2	Low–moderate	Risk stratification; not for isolated use	RCTs of HRV-guided prediction and intervention
Baroreflex arm (BRS)	Low BRS → post-induction hypotension; −35% intraoperatively in neurosurgery (21, 25)	Prosp. cohort; pilot	2–3	Low	Anticipating haemodynamic instability	Validated thresholds; direct relationship with POD
Cholinergic anti-inflammatory reflex (vagal–immune axis)	Vagal tone modulates inflammation; non-linear relationship (10, 42)	Mechanistic; observational	5	Low (mechanistic)	Rationale for vagal modulation	Confirmation in perioperative humans
Neuroinflammation/biomarkers (NfL, GFAP, IL-6)	Elevated NfL and GFAP in POD; correlation with severity (7)	SR+MA; observational	1–3	Moderate (association)	Biomarkers of injury; not causation	Causality studies and temporal windows
Cortical integration (EEG)	Low frontal α power, spindles and emergence trajectories → POD (56, 70)	SR+MA; prosp. Cohort	1–3	Moderate	Titration guided by raw EEG	Prevention RCTs with multimodal EEG
Therapeutic vagal modulation (taVNS)	taVNS reduces POD versus sham (50)	RCT	2	Emerging	Promising non-pharmacological intervention	Multicentre replication; optimal parameters

Evidence is graded with the Oxford Centre for Evidence-Based Medicine (OCEBM) (20) Levels of Evidence; “current certainty” reflects the authors’ conservative appraisal. α, frontal alpha power; BRS, baroreflex sensitivity; DFAα1, short-term detrended fluctuation analysis scaling exponent; EEG, electroencephalography; GFAP, glial fibrillary acidic protein; HF, high-frequency power; HRV, heart rate variability; IL-6, interleukin-6; MA, meta-analysis; NfL, neurofilament light chain; POD, postoperative delirium; Prosp., prospective; RCT, randomised controlled trial; SD2/SD1, Poincaré-plot variability ratio; SR, systematic review; taVNS, transcutaneous auricular vagus nerve stimulation.

**Table 5:** Key studies on autonomic dysfunction, intraoperative EEG and perioperative neurocognitive outcomes.

Author/Year	Design/population	Intervention/exposure	Comparator	Primary outcome	Limitations	Level of evidence (20)
Patel et al. (2024) (16)	SR+MA; ICU and surgery	Exposure: preoperative HRV (SDNN, RMSSD, LF, HF, LF/HF)	Not applicable (SR+MA of observational studies)	Association between HRV and POD/POCD	High heterogeneity; no universal HRV thresholds.	1 (SR+MA)
Roberts et al. (2025) (40) (HiP)	Prosp. cohort; 7-day home HRV (wearable); non-cardiac surgery ≥65 y	Exposure: 7-day home HRV (DFAα1, SD2/SD1, HF)	Patients with POD vs. without POD	Incidence of POD	Early obs.; external validation pending.	2 (prosp. cohort)
Frandsen et al. (2022) (13)	SR without MA (63 studies); major surgery	Exposure: preoperative HRV (total power, DFAα1)	Not applicable (SR without MA)	Postoperative hypotension and atrial fibrillation	Methodological variability; limited standardisation.	2 (SR)
Cui et al. (2026) (21)	Prosp. cohort; older adults, elective non-cardiac surgery, GA	Exposure: preoperative BRS	Not applicable (observational cohort)	Post-induction hypotension	No direct evidence for POD; clinical impact to be confirmed.	2 (prosp. cohort)
Zipfel et al. (2025) (25)	Pilot cohort; intracranial neurosurgery	Monitoring: continuous intraoperative BRS	Not applicable (pilot cohort)	Intraoperative variation in BRS; bradycardia	Single-centre; young population; no POD/cognition assessment.	3 (pilot)
Markus et al. (2025) (58)	Prosp. cohort; major surgery, GA	Exposure: burst suppression by hypnotic agent	Sevoflurane vs. propofol (TIVA)	POD according to burst suppression–agent interaction	Single-centre; wide CIs; external validation pending.	3 (prosp. cohort)
Obert et al. (2025) (70); Hesse et al. (2019) (54)	Prosp. cohort; elective surgery, GA	Exposure: emergence EEG trajectories (delta, spindles, α)	Not applicable (observational cohort)	POD in the PACU	Subanalysis; delirium only in the PACU; no external validation.	3 (prosp. cohort)
Xu et al. (2025) (56)	SR+MA; elective surgery	Exposure: frontal α power and intraoperative EEG	Not applicable (SR+MA)	POD	EEG heterogeneity; observational evidence.	1 (SR+MA)
Reimer et al. (2017) (15)	Prosp. obs.; major abdominal surgery	Exposure: intraoperative autonomic reactivity	Not applicable (observational cohort)	Need for vasoactive support and complications	Small sample; indirect relevance to POD.	4 (small obs.)
Yang et al. (2025) (62)	RCT; older adults, major surgery	Intervention: guided multimodal monitoring (EEG + nociception + ANS)	Standard perioperative care	Functional connectivity (fMRI) and cognitive recovery at day 7	No significant ↓ in POCD; short follow-up.	2 (RCT)
Shi et al. (2026) (50)	Multicentre double-blind RCT; ≥65 y, major non-cardiac surgery	Intervention: active taVNS (25 Hz)	Sham stimulation	Incidence of POD	Stimulation parameters and mechanisms: validation pending.	2 (RCT)
Lozano-Vicario et al. (2023) (7)	SR+MA; at-risk older adults	Exposure: NfL and GFAP biomarkers	Not applicable (SR+MA)	POD and its severity (NfL, GFAP)	Heterogeneity; association, not causation; possible publication bias.	1 (SR+MA)

GA, general anaesthesia; BHI-A, brain–heart–immune axis; BRS, baroreflex sensitivity; BS, burst suppression; cx., surgery; POD, postoperative delirium; POCD, postoperative cognitive dysfunction; RCT, randomised controlled trial; EEG, electroencephalography; AF, atrial fibrillation; GFAP, glial fibrillary acidic protein; HiP, HiPPIE study; CI, confidence interval; IL-6, interleukin-6; MA, meta-analysis; ASM, autonomic state marker; MMon, multimodal brain monitoring; ARM, autonomic reserve marker; NfL, neurofilament light chain; nocicep., nociception; NS, non-significant; obs., observational; OR, odds ratio; periop., perioperative; prosp., prospective; fMRI, functional magnetic resonance imaging; SR, systematic review; Se, sensitivity; sevo, sevoflurane; ANS, autonomic nervous system; Sp, specificity; taVNS, transcutaneous auricular vagus nerve stimulation; TIVA, total intravenous anaesthesia; ICU, intensive care unit; PACU, post-anaesthesia care unit; HRV, heart rate variability.

**Table 6:** Perioperative interventions for autonomic assessment and brain protection, organised by readiness for clinical use.

Readiness for clinical use	Interventions	Basis of evidence (20)	Key references
Currently recommended care (guideline-supported)	Preoperative cognitive and frailty assessment Medication review Multicomponent non-pharmacological prevention Adequate pain treatment Avoidance of unnecessary psychoactive medication Haemodynamic and oxygenation management – Repeated postoperative delirium screening	Supported by clinical practice guidelines and consensus (OCEBM 1–2).	(6, 17, 71)
Potentially useful but incompletely established	Selected use of dexmedetomidine EEG-informed hypnotic titration Nociception-guided analgesia – Individualised blood-pressure management	Mixed or conflicting randomised and observational evidence; not established for POD prevention (OCEBM 2–3).	(18, 21, 54, 56, 58, 62, 66, 70)
Experimental or research only	DFAα1- or SD2/SD1-guided treatment Serial ANI as a prehabilitation biomarker BRS-directed MAP targets taVNS as routine prophylaxis HRV biofeedback for POD prevention – Continuous postoperative HRV as a measure of cognitive recovery	Single small trials, protocols, or mechanistic/observational data only; not for routine clinical use (OCEBM 3–5).	(21, 25, 40, 47, 49, 50)

Interventions are grouped by their readiness for clinical use rather than by perioperative phase, so that guideline-supported care is not presented alongside experimental strategies. The basis of evidence is graded with the Oxford Centre for Evidence-Based Medicine (OCEBM) (20) Levels of Evidence. Placement in the “potentially useful” tier does not imply proven prevention of postoperative delirium; for EEG-informed titration in particular, two large randomised trials were negative for delirium. ANI, Analgesia Nociception Index; BRS, baroreflex sensitivity; DFAα1, short-term detrended fluctuation analysis scaling exponent; EEG, electroencephalography; HRV, heart rate variability; MAP, mean arterial pressure; OCEBM, Oxford Centre for Evidence-Based Medicine; POD, postoperative delirium; SD2/SD1, Poincaré-plot variability ratio; taVNS, transcutaneous auricular vagus nerve stimulation.

### 2.2 Scope of the review

The review focuses on adult patients undergoing surgery, especially those of advanced age or with frailty undergoing major non-cardiac surgery, cardiac surgery and intracranial neurosurgery. Likewise, studies from the intensive care setting were considered exclusively when they provided mechanistic evidence relevant to understanding the pathophysiological mechanisms of perioperative delirium.

## 3 Surgical stress and the autonomic nervous system

Surgical trauma triggers a neuroendocrine stress response that involves the hypothalamic–pituitary–adrenal axis, the sympathetic system and the systemic inflammatory response. The resulting autonomic dysregulation—characterised by sustained sympathetic dominance, reduced vagal tone and loss of HRV—compromises the CAP, facilitated by the vagus nerve, which under physiological conditions limits the systemic inflammatory response and its translation to the central compartment (16). When vagal tone is chronically reduced, the CAP operates with reduced capacity, allowing the inflammatory cascade to reach critical thresholds of microglial activation. Perioperative haemodynamic perturbation—post-induction hypotension, episodes of cerebral hypoperfusion—represents an additional pathophysiological mechanism of acute brain injury (21).

Haemodynamic instability may play an important role in susceptible patients. Post-induction hypotension, intraoperative hypotension, impaired autoregulation, anaemia and hypoxaemia may reduce oxygen delivery to vulnerable cerebral regions (14, 17, 22). Older adults with cerebrovascular disease, frailty or autonomic dysfunction may be less able to maintain stable cerebral perfusion during anaesthetic induction and surgical stress.

Moreover, major surgery, the cerebral response, haemodynamic instability and perioperative autonomic dysfunction, including baroreflex impairment and intraoperative vegetative responses, are associated with an increased risk of POD (21, 23–25).

The ANS regulates bodily homeostasis through the dynamic coordination of cardiovascular, respiratory, metabolic, thermoregulatory and immune responses (12, 14). In the perioperative period, the ANS is continuously challenged by surgical stress, as we have noted (13, 14, 26).

This complex response cannot be fully captured by isolated measurements of heart rate or blood pressure. Two patients may have similar values of mean arterial pressure but very different autonomic states, baroreflex function, inflammatory responses and cerebral vulnerability. Autonomic monitoring may therefore provide complementary information about the patient’s capacity to adapt to perioperative stress.

The vagus nerve is a fundamental component of parasympathetic regulation and contributes to the interaction between the brain, the heart and the immune system. Vagal signalling participates in a proposed neuroimmune circuit that modulates cytokine release but the precise anatomical relay remains debated (10, 12, 27–29).

Numerous factors shift the sympathetic–parasympathetic balance towards sustained sympathetic dominance, which may bring about a dysregulation of the brain–heart–immune axis. The reduction of vagal modulation limits the cholinergic anti-inflammatory reflex, favouring systemic inflammation, blood–brain barrier dysfunction and neuroinflammation implicated in POD (12, 14, 30). The capacity to maintain this balance constitutes systemic autonomic resilience.

The autonomic nervous system (ANS) occupies a mechanistically central position in the physiological vulnerability of the older surgical patient. The concept of allostatic load (31) offers one of the most robust mechanistic bridges between that reduction of reserve and the decline in autonomic complexity: the cumulative wear upon the regulatory systems—cardiovascular, neuroendocrine, immune and metabolic—translates into a sustained reduction of the fractal variability of the R-R intervals, with preferential loss of the adaptability indices (DFAα1, SD2/SD1) before the state markers (SDNN, RMSSD) (32, 33). With the passage of time, changes in the sinoatrial node reduce the capacity to generate intrinsic variability of the cardiac rhythm (34), and individuals with lower reserve consistently show diminished RMSSD, SDNN and high-frequency power relative to age-matched controls (32). Rather than the mere decline of baseline autonomic tone, it is the loss of parasympathetic modulation and of physiological adaptability that proves relevant to POD risk. This reading accords with the evidence derived from critical illness: a low SDNN is associated with the severity of the multiple organ dysfunction syndrome and with in-hospital mortality (35), and in septic shock the ANS appears to progress from adaptive dysregulation to an almost complete autonomic paralysis in the most severe cases (30).

### 3.1 Heart rate variability

HRV refers to the analysis of the temporal fluctuations between successive normal beats, usually derived from the R-R intervals, and reflects the dynamic interaction between the sympathetic, parasympathetic, respiratory, baroreflex and central mechanisms of regulation (13, 32, 33). HRV is usually analysed by means of time-domain, frequency-domain and non-linear methods.

The time-domain indices include the standard deviation of the NN intervals (SDNN) and the root mean square of successive differences (RMSSD). RMSSD is frequently interpreted as a marker strongly influenced by parasympathetic modulation, although it is also affected by respiration and the recording conditions. The frequency-domain indices include the low-frequency (LF) and high-frequency (HF) components (36). High-frequency power is closely related to respiratory sinus arrhythmia and parasympathetic modulation, whereas low-frequency power reflects mixed sympathetic, parasympathetic and baroreflex influences. The LF/HF ratio must therefore be interpreted with caution and cannot be regarded as a simple measure of sympatho-vagal balance.

The non-linear HRV metrics, such as SD1, SD2, entropy-based measures and detrended fluctuation analysis, may provide additional information about physiological complexity and adaptive capacity.

By virtue of its dynamic character, heart rate variability (HRV) does not quantify frailty directly, but rather the autonomic substrate upon which it exerts its greatest perioperative impact (37, 38). In this context, preoperative autonomic assessment by means of HRV should be understood as a complement, and not as a substitute, for comprehensive clinical evaluation. Its principal contribution lies in providing a dynamic physiological measure of biological resilience and of the organism’s capacity to respond to surgical stress. Thus, HRV may help to identify patients with lower adaptive reserve, independently of their chronological age (32, 33). Its interpretation must always be contextualised, because the same value may signify different things according to age, comorbidity, cognitive status or pharmacological exposure (13, 16, 33).

Non-linear HRV indices provide complementary information on the temporal organization and complexity of cardiac interbeat dynamics. DFAα1 is the short-term scaling exponent derived from detrended fluctuation analysis and characterises fractal correlation properties over short time scales; it should not be described as a measure of long-range temporal dependence. The SD2/SD1 ratio is derived from Poincaré plot geometry and expresses the relationship between longer-term and short-term variability within the analysed recording, but it has not been validated as a direct measure of sympathovagal flexibility, autonomic reserve, or adaptability. In this review, the distinction between conventional “state” measures, such as SDNN, RMSSD, HF, and LF, and proposed “adaptability” measures, such as DFAα1 and SD2/SD1, is therefore presented explicitly as an interpretive framework rather than an established HRV taxonomy (13).

This framework is intended to distinguish indices that predominantly describe autonomic cardiac dynamics within a specific measurement period from indices hypothesized to reflect aspects of temporal organization or responsiveness to physiological perturbation; however, the empirical support for these assignments remains limited. Moreover, HF power and RMSSD are influenced by respiratory rate and tidal volume and should not be equated uncritically with vagal tone, whereas LF reflects multiple physiological influences and cannot be interpreted as a specific marker of sympathetic activity. Accordingly, the LF/HF ratio should not be considered a direct measure of sympathovagal balance, and an increased ratio alone does not demonstrate sustained sympathetic predominance. More broadly, HRV is a cardiac-output signal shaped by autonomic, respiratory, haemodynamic, behavioural, and methodological factors; it does not directly measure central vagal activity, the cholinergic anti-inflammatory pathway, or the functional integrity of the entire brain–heart–immune axis (Tables 1–3).

### 3.2 Baroreflex sensitivity

BRS quantifies the reflex relationship between changes in blood pressure and compensatory changes in heart rate. It reflects the capacity of the cardiovascular system to buffer fluctuations in blood pressure and preserve organ perfusion (22, 39). In the perioperative context, impaired BRS may identify patients at greater risk of post-induction hypotension, exaggerated haemodynamic instability and, potentially, unstable cerebral perfusion (22).

BRS may be assessed by means of continuous recordings of blood pressure and electrocardiography, although fully non-invasive methods with photoplethysmography and continuous digital blood pressure monitoring are being increasingly studied. As with HRV, BRS is influenced by age, medications, hydration status, anaesthetic depth, vascular stiffness and comorbidities.

Despite their potential usefulness, HRV and BRS are subject to methodological limitations that condition their interpretation. Numerous clinical and pharmacological factors act simultaneously upon the autonomic indices: age, arrhythmias—in particular atrial fibrillation—and pacemakers, beta-blockers, anticholinergic drugs, antidepressants and opioids, the ventilatory mode and pattern, sleep quality, pain, anxiety and the very duration of the recording (13, 39). Since, moreover, perioperative conditions differ substantially from controlled laboratory settings, the same value may carry different meanings according to the circumstances, and any proposed threshold must be regarded as indicative and context-dependent, never a universal and definitive cut-off point.

### 3.3 The brain–heart–immune axis

The brain–heart–immune axis offers a conceptual framework for understanding how autonomic dysfunction may be linked to a neuropsychiatric complication such as POD (10, 12, 40). Within it, vagal signalling figures among the neurobiological mechanisms that regulate peripheral innate immune activation following surgical injury: the cholinergic anti-inflammatory pathway (CAP) describes the reflex by which vagal activity modulates cytokine release and the inflammatory response (10, 12). When surgical trauma activates the peripheral immune pathways, the patient with preserved autonomic adaptability maintains that reaction as proportionate and self-limiting; by contrast, frailty, reduced HRV, impaired BRS or chronic inflammation diminish the regulatory capacity of the axis (32–34).

The model proposed by Acker et al. (12) integrates three physiologically distinct but interconnected arms. The autonomic arm links the regulatory capacity of the central nervous system with cardiovascular function through the sympathetic and parasympathetic branches, and determines HRV, BRS and haemodynamic stability. The neuroimmune arm connects autonomic activity with systemic inflammation by means of the CAP: the vagal efferents release acetylcholine at the splenic nerve–spleen junction, activate the *α*7nAChR receptors of the macrophages and inhibit the production of pro-inflammatory cytokines (TNF-α, IL-1β, IL-6) by suppressing NF-κB (10, 41). The neurocognitive arm closes the loop: the neuroinflammation that traverses a vulnerable blood–brain barrier triggers cascades of microglial activation that converge upon POD.

It should be specified that the relationship between vagal tone and immune response is not unidirectional. Lammers-Lietz et al. (42) showed that an elevated vagal tone may, paradoxically, mediate perioperative immunosuppression through the CAP and raise the risk of postoperative infection. The therapeutic objective is therefore not to maximise vagal tone, but to restore functional balance and adaptive capacity, prioritising the adaptability indices (DFAα1, SD2/SD1) over the static markers of tone (RMSSD, HF power); both its excessive suppression and its excessive activation reflect dysregulation rather than a calibrated response.

**Figure 1:**
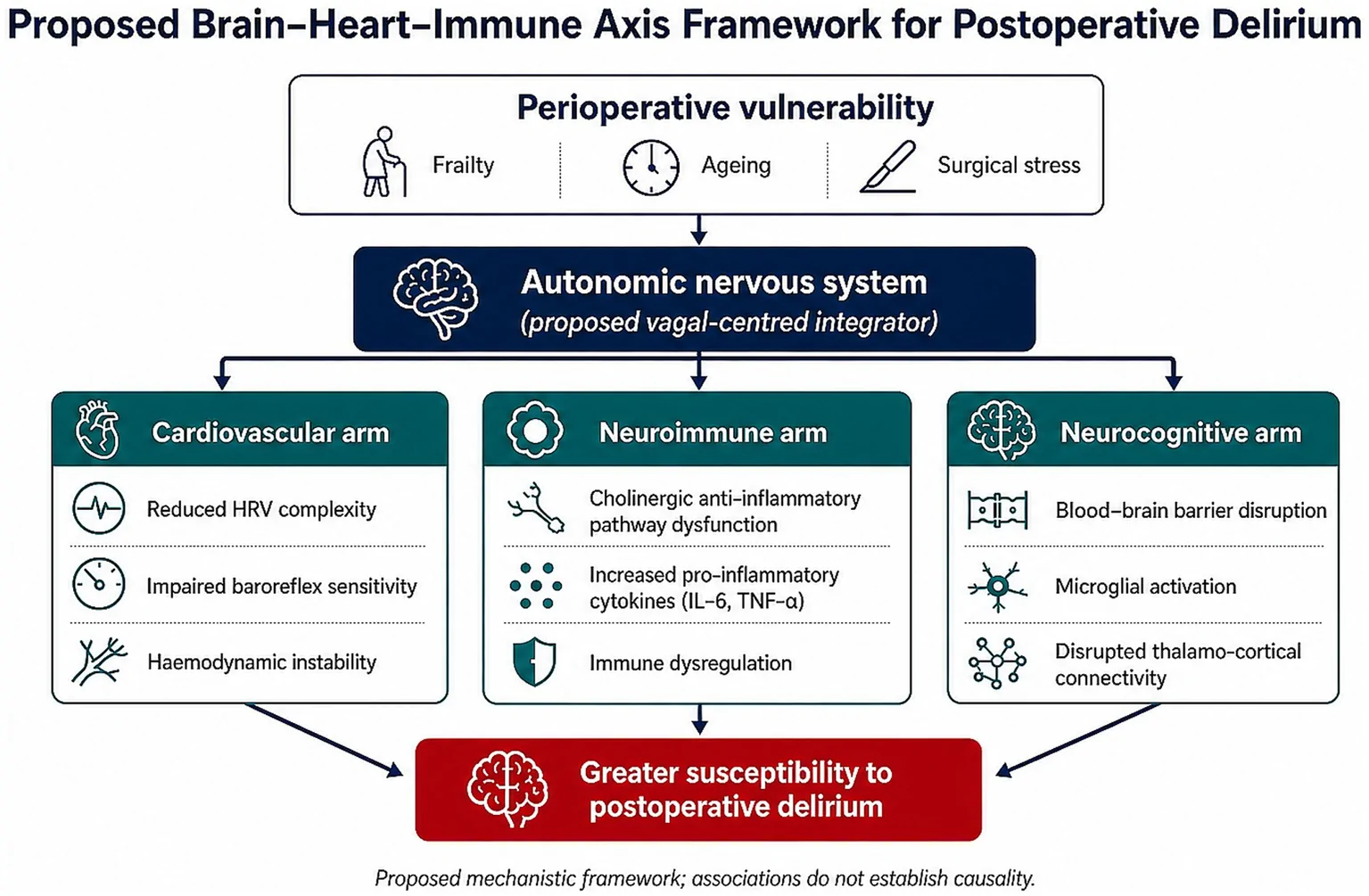
Proposed brain–heart–immune axis framework for susceptibility to postoperative delirium. Frailty, ageing, and surgical stress may converge on autonomic dysregulation. Within this hypothesis-generating, vagal-centred framework, altered cardiovascular regulation, neuroimmune signalling, and neurocognitive vulnerability are conceptualised as interacting pathways that may increase susceptibility to postoperative delirium (POD). External validation is required before these indices can be incorporated into clinical prediction or decision-making. Arrows represent proposed relationships and do not establish causality. BRS, baroreflex sensitivity; HRV, heart rate variability; IL-6, interleukin-6; POD, postoperative delirium; TNF-*α*, tumour necrosis factor alpha.

These pathways converge upon the blood–brain barrier and the neurovascular unit, where they promote neuroinflammatory signalling, microglial activation, altered neurotransmission and network dysfunction (4, 5, 7) (Figure 1).

This model does not attribute POD to a single cause: it proposes that autonomic deterioration acts as a measurable physiological bridge between preoperative vulnerability and perioperative triggers, and that its markers may help to identify the patients in whom surgical stress will exceed adaptive capacity. So as not to go beyond what the evidence sustains, the axis must be understood as an integrative conceptual framework, supported by heterogeneous levels—above all observational, mechanistic and of physiological plausibility—and not as a validated clinical model; a good part of that evidence derives, moreover, from contexts foreign to the perioperative setting or not specific to POD, so that its translation is uncertain. Autonomic resilience is proposed here as a unifying hypothesis, not as a demonstrated causal mechanism.

Table 4 details, for each component, the principal evidence, its design, the OCEBM level, the current certainty, the clinical implications and the research priorities, its validation will require prospective studies designed specifically in the surgical population.

## 4 Postoperative delirium and its relationship with autonomic dysfunction

### 4.1 Preoperative HRV and POD

The systematic study of the relationship between HRV and POD has generated a body of work that is modest in scale but conceptually relevant. In perioperative and intensive care populations, altered HRV indices have been associated with delirium and other adverse outcomes, although heterogeneity remains substantial (16, 35, 40) Table 5.

The first systematic review with meta-analysis designed to synthesise this association (16) included 9 studies (*n* = 785; published between 2015 and 2023) in intensive care and major surgery, and showed that SDNN displayed the most robust and consistent association with the presence or development of delirium. The RMSSD, LF and HF indices and the LF/HF ratio showed weak or absent associations, although methodological heterogeneity limited interpretation.

An apparent paradox warrants pathophysiological attention. Whilst some studies, particularly in intensive care, found higher values of SDNN, RMSSD and entropy in patients with delirium, other studies in the surgical setting found the inverse association, with reduced HRV predicting a greater risk of POD (43), in 75 patients with hip fracture, found higher values of SDNN and HF in patients with delirium; Sun et al. (44), in 294 older patients undergoing orthopaedic surgery, identified negative correlations between SDNN and POD. This coexistence of associations in both directions can be partly reconciled through the state/adaptability framework: in severe critical illness, elevated SDNN may reflect autonomic hyperactivation and loss of tonic inhibition (a maladaptive excitatory dysregulation); in surgical frailty, reduced HRV reflects adaptive insufficiency. Both extremes represent forms of dysregulation that demand a context-specific interpretation.

The prospective HiPPIE study (40) included 139 patients aged ≥65 years undergoing major non-cardiac, non-intracranial surgery and used wrist-worn devices to obtain nocturnal HRV measurements during the 7 days preceding surgery. In adjusted analyses, lower DFAα1 (adjusted OR 0.47; 95% CI 0.26–0.79; *p* = 0.007) and SD2/SD1 (adjusted OR 0.44; 95% CI 0.23–0.78; *p* = 0.008), as well as higher peak HF (adjusted OR 1.89; 95% CI 1.17–3.18; *p* = 0.012), were associated with POD. Higher preoperative SD2/SD1 and DFAα1 values were also associated with smaller postoperative increases in IL-6 (SD2/SD1 × time: *β* = −0.22; 95% CI − 0.37 to −0.06; *p* = 0.009; DFAα1 × time: β = −0.25; 95% CI − 0.39 to −0.10; *p* = 0.002). These findings suggest a potential relationship between preoperative autonomic adaptability, POD susceptibility, and the postoperative IL-6 trajectory; however, they do not demonstrate that autonomic adaptability modulates inflammation or confirm the proposed mechanistic sequence of the BHI-A framework. HiPPIE should therefore be regarded as an exploratory, single-centre observational study whose results are hypothesis-generating and potentially subject to residual confounding, rather than as definitive evidence that adaptability indices are superior predictors of POD.

The systematic review by Frandsen et al. (13), which included 63 studies examining preoperative HRV and perioperative outcomes, provides complementary evidence that reduced autonomic variability may be associated with adverse perioperative outcomes: low preoperative total power was associated with post-induction hypotension, whereas DFAα1 below 1.05 predicted postoperative atrial fibrillation in three of the four studies assessing this measure. Nevertheless, the observation that DFAα1 is associated with different cardiovascular and neurocognitive outcomes does not by itself validate it as a universal marker of perioperative reserve. Overall, systematic reviews, meta-analyses, and recent wearable-based studies support an association between reduced HRV complexity, diminished parasympathetic modulation, impaired autonomic adaptability, and greater POD susceptibility (13, 16, 40). These findings are consistent with—but do not prove—the hypothesis that preoperative brain–heart–immune dysregulation may reduce tolerance to perioperative inflammatory and haemodynamic stress. Substantial heterogeneity in study populations, HRV acquisition and analytical methods, wearable technologies, and delirium assessment, together with the observational nature and limited sample size of the available evidence, underscores the need for standardised, adequately powered multicentre validation studies.

### 4.2 BRS and haemodynamic instability

Impairment of BRS may contribute to perioperative vulnerability by reducing the patient’s capacity to buffer changes in arterial pressure (22, 39). Post-induction hypotension is frequent in older adults and may be particularly relevant in patients with impaired autonomic compensation. Reduced BRS may therefore identify patients at risk of haemodynamic instability during anaesthetic induction.

Although the direct relationship between BRS and POD requires further investigation, the mechanistic link is plausible. Impaired baroreflex function may predispose to post-induction hypotension, cerebral hypoperfusion and impaired cerebral oxygen delivery in vulnerable patients (14, 22). In vulnerable brains, even transient haemodynamic instability may contribute to postoperative cognitive dysfunction when combined with inflammation, pain, sleep disturbance and exposure to sedatives.

Impairment of baroreflex sensitivity (BRS) may contribute to perioperative vulnerability by reducing the body’s capacity to buffer fluctuations in arterial pressure and maintain haemodynamic stability in the face of anaesthetic-surgical stress (22, 39). In this context, post-induction hypotension—a frequent event in older adults—may acquire particular relevance in patients with limited autonomic reserve. Accordingly, reduced BRS could identify individuals at greater risk of haemodynamic instability during anaesthetic induction. Although the direct association between diminished preoperative BRS and postoperative delirium (POD) has not been demonstrated causally in clinical studies, there exists a plausible pathophysiological sequence supported by physiological evidence: reduced BRS is associated with greater susceptibility to post-induction hypotension (21, 22), which could favour episodes of cerebral hypoperfusion and disturbances in cerebral oxygen delivery in patients with compromised autoregulatory mechanisms (14). In vulnerable brains, these transient haemodynamic disturbances could act synergistically with other perioperative factors, such as neuroinflammation, pain, sleep disturbance, exposure to sedatives and blood–brain barrier dysfunction, favouring the onset of POD. However, this mechanistic chain remains principally inferential and requires prospective validation through studies that simultaneously assess preoperative baroreflex function, intraoperative haemodynamic stability and postoperative neurocognitive outcomes.

### 4.3 Pathophysiological mechanisms

The principal perioperative autonomic disturbances include diminished baroreflex sensitivity, reduced HRV complexity and sustained sympathetic predominance. Baroreflex dysfunction favours post-induction hypotension and cerebral hypoperfusion (21, 25), whilst sympathetic hyperactivation reduces the efficacy of the cholinergic anti-inflammatory reflex, potentiates the neuroinflammatory response and is associated with greater organ and neurocognitive dysfunction (14, 45). Likewise, alteration of the autonomic modulation of the circadian rhythm contributes to the disruption of sleep and of glymphatic function, favouring the onset of delirium (4). These findings demonstrate that conventional haemodynamic monitoring does not by itself reflect the perioperative cerebral physiological state.

**Figure 2:**
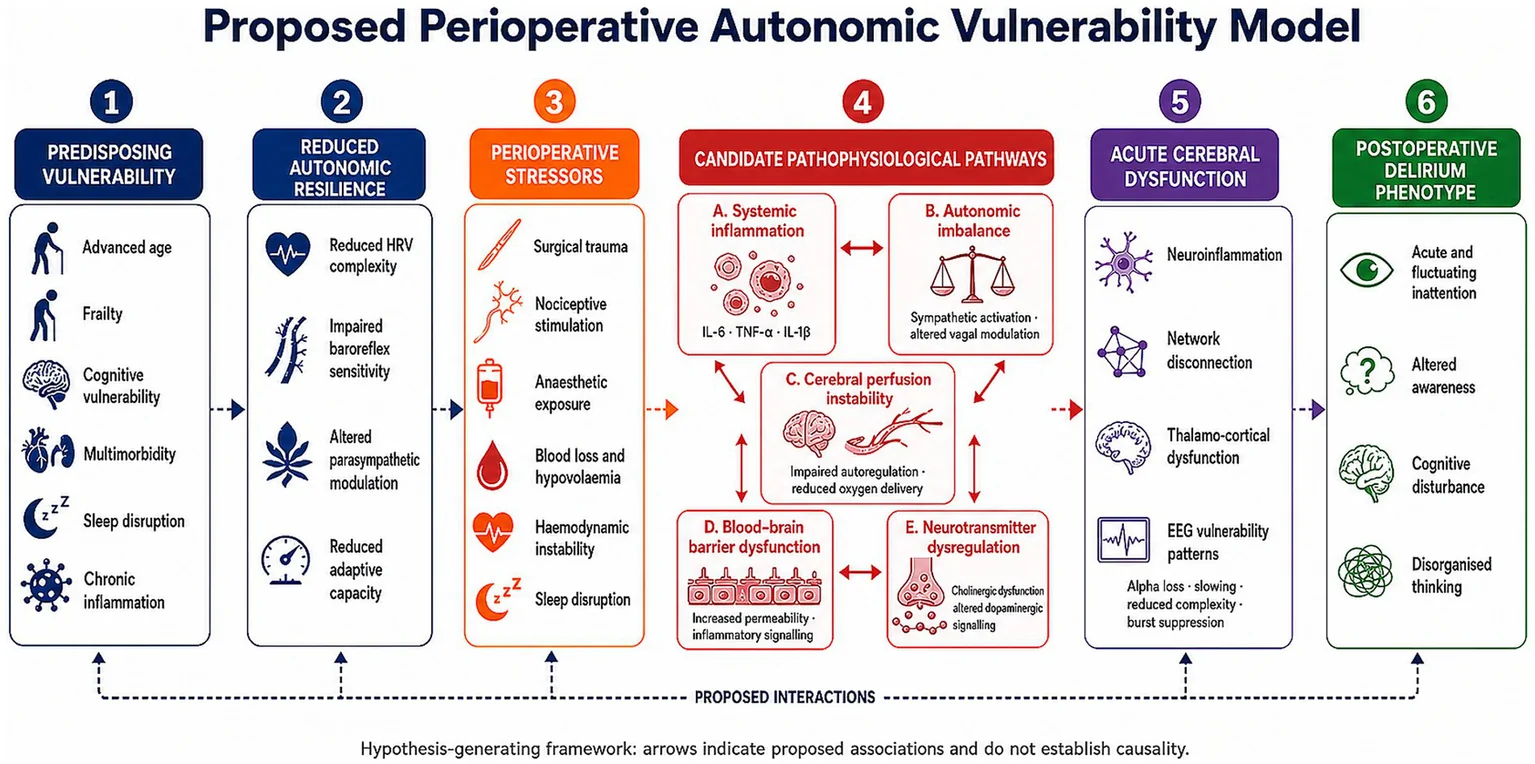
Proposed perioperative autonomic vulnerability model for postoperative delirium. Predisposing vulnerability and reduced autonomic resilience may interact with perioperative stressors to increase susceptibility to acute cerebral dysfunction and postoperative delirium (POD). This figure represents a hypothesis-generating conceptual framework: arrows indicate proposed associations and interactions and do not establish causality. EEG, electroencephalography; HRV, heart rate variability; IL-1*β*, interleukin-1 beta; IL-6, interleukin-6; POD, postoperative delirium; TNF-α, tumour necrosis factor alpha.

The available evidence supports the incorporation of preoperative autonomic assessment into multimodal POD risk stratification. It is an accessible, low-cost tool, applicable by means of ECG or photoplethysmography, even in patients with substantial functional limitation (13). Its interpretation must be undertaken together with clinical assessment, cognitive screening and other biomarkers, since no single marker offers sufficient predictive capacity Figure 2.

## 5 Prehabilitation and modulation of the autonomic nervous system

### 5.1 Prehabilitation

The multimodal prehabilitation framework, aligned with Enhanced Recovery After Surgery (ERAS) principles, provides an operational structure integrating exercise, nutritional optimization, correction of anaemia, psychological support, sleep optimization, and management of comorbidities. Within this framework, the autonomic nervous system may represent a physiological axis linking preoperative conditioning with intraoperative and postoperative resilience (37, 46). From an autonomic perspective, the aim of prehabilitation is to enhance adaptive capacity rather than achieve a predefined absolute HRV value. Serial changes in exploratory measures such as DFAα1 or SD2/SD1 may help characterise individual autonomic trajectories during a prehabilitation programme (13, 47).

However, no outcome trial has demonstrated that increasing DFAα1 or SD2/SD1 represents a validated therapeutic target or surrogate endpoint for reducing postoperative delirium (POD). These measures should therefore be interpreted as hypothesis-generating indicators rather than established treatment goals. Similarly, although prehabilitation may be associated with changes in cardiovascular adaptability, respiratory efficiency, inflammatory regulation, sleep quality, and stress tolerance, these mechanisms have not been demonstrated as mediators of POD prevention. In particular, nutritional optimization has not been shown to improve blood–brain barrier integrity in a clinically measurable manner. The ongoing PreANI study is a protocol investigating whether HRV-derived autonomic metrics can detect functional changes during multimodal prehabilitation and whether such changes are associated with postoperative trajectories; it does not validate the Analgesia Nociception Index for serial frailty assessment or provide evidence of intervention efficacy.

### 5.2 Aerobic exercise and resistance training

Aerobic exercise has the most consistent evidence for improving HRV and parasympathetic modulation. Regular moderate-to-vigorous physical activity has been associated with increased SDNN and RMSSD, improved baroreflex sensitivity, reduced resting heart rate, and attenuation of systemic inflammation (13, 14). Resistance training may complement aerobic exercise by improving muscle mass, mitochondrial function, and insulin sensitivity, thereby potentially reducing the metabolic and inflammatory burden associated with impaired physiological reserve (48). For patients with substantial functional limitations, adapted chair-based exercise, respiratory training, and progressive mobilisation may provide incremental benefits within appropriate safety constraints. Nevertheless, these physiological effects should not be interpreted as evidence that exercise-induced autonomic or inflammatory changes mediate POD prevention, because this causal pathway has not been established in clinical outcome trials.

### 5.3 HRV biofeedback and respiratory resonance

Respiratory HRV biofeedback is a potential prehabilitation intervention based on paced breathing, frequently near the individual resonance frequency, to enhance cardiorespiratory coupling and baroreflex engagement. Although this approach may increase HRV and improve autonomic regulation in selected populations, its perioperative application remains investigational, and there is currently no evidence that HRV biofeedback prevents POD. In a narrativa review, Ramakumar and Sama (49) provides a conceptual synthesis rather than evidence of intervention efficacy. For research purposes, an “autonomic frailty phenotype” may be operationally defined as a multidimensional pattern characterised by reduced autonomic variability and complexity, impaired baroreflex function, and blunted physiological responsiveness or recovery following stress. However, this construct has no validated diagnostic thresholds and should not be presented as an established clinical diagnosis. Likewise, a “documented cholinergic deficit” cannot currently be established directly in routine perioperative practice and may only be inferred indirectly from convergent autonomic, inflammatory, and clinical findings. Accordingly, the proposition that preoperative transcutaneous auricular vagus nerve stimulation “primes” the cholinergic anti-inflammatory pathway remains hypothetical, and neither HRV biofeedback, serial autonomic adaptability targets, nor taVNS-mediated CAIP priming should currently be considered established strategies for POD prevention.

### 5.4 Transcutaneous auricular vagus nerve stimulation (taVNS)

Recognition that baseline physiological reserve influences postoperative outcomes has increased interest in prehabilitation, which encompasses interventions undertaken before surgery to improve functional capacity and resilience, including exercise, nutritional optimization, correction of anaemia, psychological support, sleep optimization, and management of comorbidities (37, 46). Autonomic modulation through heart rate variability biofeedback or transcutaneous auricular vagus nerve stimulation (taVNS) has been proposed as an additional component of multimodal prehabilitation for selected high-risk patients. TaVNS is an experimental, non-invasive approach intended to modulate autonomic and inflammatory pathways. In a sham-controlled randomized trial involving 150 patients, intraoperative auricular vagus nerve stimulation was associated with a lower incidence of postoperative delirium (POD) in the active-treatment group (50). Although encouraging, this finding derives from a single relatively small trial and requires confirmation in adequately powered multicentre studies. Furthermore, the reported mediation analysis involving postoperative sleep quality was exploratory and does not establish a causal vagal–thalamo–cortical–sleep mechanism.

Ongoing multicentre protocols (41, 51), mechanistic hypotheses (52, 53), and preclinical studies provide a scientific rationale for further investigation but should not be considered evidence of clinical efficacy. Neither taVNS nor HRV biofeedback currently has sufficient replicated evidence to support a universal perioperative recommendation. Before routine adoption, future studies should standardise stimulation frequency, intensity, duration, and timing relative to surgery; define appropriate patient-selection criteria and the frailty phenotypes most likely to benefit; clarify the underlying mechanisms; and establish long-term safety and clinical reproducibility.

## 6 Intraoperative multimodal monitoring

Standard intraoperative monitoring includes heart rate, arterial pressure, oxygen saturation, capnography, temperature and neuromuscular monitoring. Although essential, these variables may be insufficient to characterise the interaction between cerebral vulnerability, nociceptive stimulus, autonomic stress and haemodynamic instability.

A multimodal monitoring approach may provide a more complete physiological picture. This includes electroencephalography for cortical activity, nociception monitoring for autonomic and brainstem responses to surgical stimulation, and advanced haemodynamic monitoring for perfusion and cardiovascular adaptability.

### 6.1 Intraoperative electroencephalographic monitoring

Current evidence suggests that electroencephalographic monitoring makes it possible to identify vulnerable brains and to facilitate a more individualised titration of hypnotics (18, 54–56). The appearance of burst suppression (BS) may reflect distinct physiological states of the cerebral cortex, including diminished cortical reserve in certain patients, and may be associated with autonomic dysfunction, with reduced vagal tone, impaired baroreflex sensitivity and alteration of the cholinergic anti-inflammatory reflex (57–59). Nevertheless, two recent large studies—ENGAGES [(60); *n* = 1,232] and ENGAGES-Canada [(61); *n* = 1,140]—reported no significant reduction in postoperative delirium. Thus, the evidence supporting an association between intraoperative burst suppression and postoperative delirium remains conflicting and of very low certainty.

The interpretation of the raw EEG and of the density spectral array (DSA) makes it possible to contextualise these physiological findings beyond the processed indices (55, 62). In conclusion, raw and processed EEG might identify anaesthetic sensitivity and potentially help avoid unnecessarily high hypnotic exposure, but large randomized trials have not consistently demonstrated that EEG guidance prevents postoperative delirium.

### 6.2 Monitoring of nociception and stress

Surgical stimulation activates nociceptive responses involving spinal, supraspinal, endocrine, and autonomic pathways, with potential effects on haemodynamics, inflammation, and postoperative recovery, even during general anaesthesia (26, 63, 64). Nociception monitors—including the Analgesia Nociception Index (ANI), Nociception Level (NOL), pupillary dilatation, skin conductance, and other multiparametric models—estimate the balance between nociceptive stimulation and antinociception using signals derived largely from autonomic nervous system activity (26, 55, 64). However, these technologies rely on different physiological signals and algorithms and should not be considered interchangeable. For example, ANI is primarily derived from heart rate variability (HRV), whereas NOL integrates multiple physiological variables. Their principal clinical purpose is to support individualized analgesic titration rather than to provide a comprehensive assessment of autonomic function.

Nevertheless, these indices may provide complementary information on intraoperative autonomic modulation, particularly in patients with reduced preoperative autonomic reserve identified using measures such as DFAα1 or baroreflex sensitivity. Within the BHI-A framework, the integration of HRV-derived information with electroencephalographic and haemodynamic monitoring offers a coherent multimodal approach: HRV provides a proxy for the autonomic component, EEG reflects the neurocognitive component, and haemodynamic variables represent the integrated physiological expression of their interaction. Although nociception-guided monitoring may facilitate more individualized analgesic administration and reduce both under- and overdosing, its measurements are influenced by numerous physiological and pharmacological factors and should therefore be interpreted within a multimodal monitoring strategy (63, 64).

Moreover, physiological responsiveness to nociceptive stimulation, changes in opioid administration, and improvements in postoperative pain or recovery should be distinguished from direct evidence of delirium prevention. Evidence supporting continuous intraoperative HRV or nociception monitoring for predicting or preventing postoperative delirium (POD) remains preliminary (25, 47).

In a single-centre randomized trial involving older patients undergoing knee arthroplasty, NOX-guided management was associated with a lower incidence of POD on postoperative day 1 than standard care (7% vs. 16%) (65). However, this finding should be interpreted cautiously given the study’s relatively small sample, procedure-specific population, single-centre design, and need for independent replication. Accordingly, nociception-guided analgesia should be regarded as a strategy for optimising the nociception–antinociception balance rather than necessarily eliminating opioid administration. Adequately powered multicentre trials are required to establish whether this approach reduces the incidence, severity, or duration of POD.

### 6.3 Advanced haemodynamic and autonomic integration

Haemodynamic management is central to neuroprotection. Mean arterial pressure alone may not fully reflect cerebral perfusion, especially in patients with impaired autoregulation, vascular stiffness, autonomic dysfunction or frailty. Advanced haemodynamic monitoring, including dynamic indices of preload responsiveness, cardiac output, perfusion markers and individualised arterial pressure targets, may help to reduce episodes of hypoperfusion. Witteveen et al. validated the ambulatory estimation of BRS by means of photoplethysmography (PPG) in comparison with reference arterial-catheter methods, supporting home-based preoperative stratification of BRS without hospital infrastructure (14, 22, 39).

Integrated monitoring may help clinicians to titrate hypnotic, analgesic and vasoactive therapies with greater precision, potentially reducing haemodynamic instability and excessive physiological stress. However, whether this strategy directly reduces the incidence of POD remains to be demonstrated in adequately powered interventional trials.

## 7 Integrated perioperative approach

**Figure 3:**
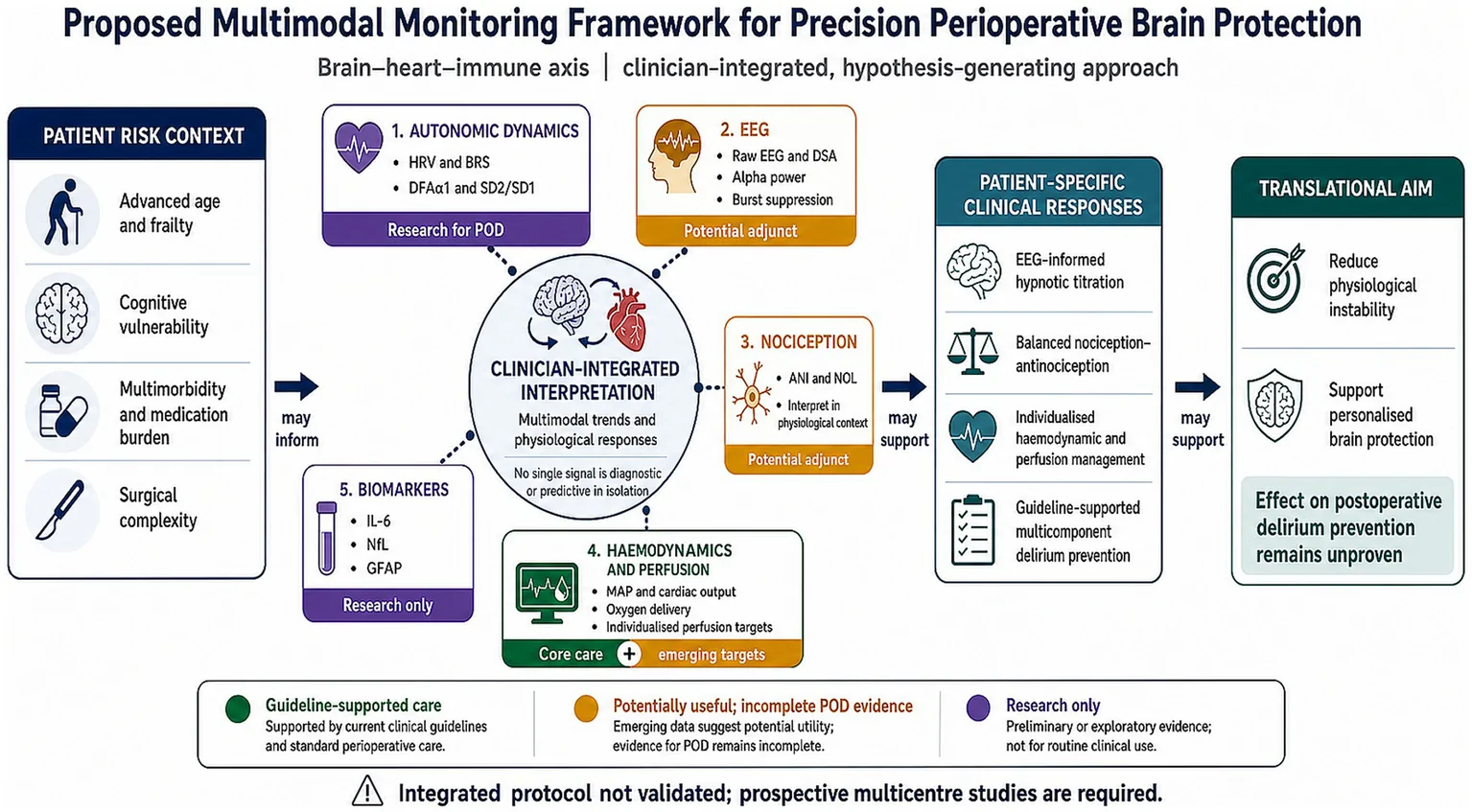
Proposed multimodal monitoring framework for precision perioperative brain protection. Clinical risk factors provide the context for clinician-integrated interpretation of perioperative physiological information. The proposed framework combines autonomic indices, electroencephalography, nociception monitoring, haemodynamic and perfusion variables, and exploratory biomarkers. Nevertheless, the complete integrated protocol has not been prospectively validated, no individual signal should be interpreted as a standalone diagnostic or predictive tool, and its effect on POD prevention remains unproven. ANI, analgesia nociception index; BRS, baroreflex sensitivity; DFAα1, short-term detrended fluctuation analysis scaling exponent; DSA, density spectral array; EEG, electroencephalography; GFAP, glial fibrillary acidic protein; HRV, heart rate variability; IL-6, interleukin-6; MAP, mean arterial pressure; NfL, neurofilament light chain; NOL, nociception level index; POD, postoperative delirium; SD2/SD1, Poincaré-plot long-to-short variability ratio.

Synthesising the evidence reviewed, we propose an integrative perioperative model in four phases—assessment, prehabilitation, multimodal monitoring and postoperative protection—in which the autonomic nervous system acts as the physiological thread that connects preoperative vulnerability with postoperative cognitive evolution (Figure 3; Table 5) (12, 14, 40). The model integrates clinical and autonomic assessment, preoperative optimisation, multimodal monitoring and the delirium-prevention strategies developed throughout this review (13, 16, 17, 25, 32–34, 39, 46, 55, 58, 63, 64, 66, 67). Although some of its components enjoy robust scientific support, the complete integrated protocol has not been validated in randomised clinical trials; it must therefore be interpreted as a conceptual framework for the investigation and development of personalised perioperative medicine strategies, rather than as an established clinical recommendation (Figure 3; Table 6).

The singular characteristic of the model is its mechanistic continuity: the same physiological dimension—autonomic adaptive capacity—is assessed preoperatively (HRV/BRS), targeted during prehabilitation (exercise/taVNS), monitored intraoperatively (EEG/ANI/nociceptive proxies) and protected postoperatively (dexmedetomidine/non-pharmacological bundles). The ANS provides not only a diagnostic window but a therapeutic thread that runs through the entire perioperative continuum.

Success criteria could be defined in each phase according to the patient’s autonomic classification, the measurement and trajectory of the DFAα1 values, the absence of burst suppression and the episodes of hypotension during surgery, or the measurement of a negative CAM scale at 48 h postoperatively. These metrics have not yet been validated as primary endpoints in randomised trials and are presented here as possible objectives for future research.

Taken together, in the vulnerable patient, surgical injury, hypovolaemia, pain, inflammation, anaesthetic exposure, sleep disturbance and haemodynamic instability impose a high physiological burden on a system whose reserve is already exhausted (13, 14, 17). In parallel, impaired baroreflex function may increase susceptibility to post-induction hypotension and cerebral hypoperfusion (10, 12, 22). In susceptible patients, these mechanisms may converge with cholinergic imbalance, alteration of thalamocortical connectivity, excessive cortical suppression, disruption of the sleep–wake cycle and postoperative pain, ultimately contributing to the clinical phenotype of POD (4, 5, 11, 18).

“Wearables” and other continuous monitoring technologies could extend autonomic assessment into the postoperative period and facilitate the early detection of physiological deterioration.

The current evidence does not support the universal implementation of systematic autonomic monitoring for the prevention of POD; its utility will therefore need to be confirmed in prospective studies with standardised protocols, predefined thresholds and clinically relevant neurocognitive outcomes.

## 8 Limitations of the current evidence

As already noted, the interpretation of heart rate variability (HRV) and baroreflex sensitivity (BRS) is constrained by substantial methodological limitations. For this reason, neither HRV nor BRS should currently be interpreted in isolation as diagnostic or predictive tools; rather, they should be integrated within a multimodal assessment that combines frailty, cognitive screening, comorbidity, medication review, haemodynamic monitoring and electroencephalographic information.

To these measurement limitations must be added those of the evidence itself. This is predominantly observational and highly heterogeneous in terms of populations, methods and recording durations—particularly for BRS, which limits causal inference (16, 68). Consistent with this, the brain–heart–immune axis must be acknowledged as a theoretical scaffold: no randomised trial has validated the complete four-phase perioperative protocol as an integrated intervention, and the evidence for the cholinergic anti-inflammatory reflex in human surgery is indirect—inferred from animal models and from systemic markers such as IL-6, that is, mechanistic rather than causal in nature. Even the HiPPIE study (40), the most robust available, retains incompletely controlled confounders, such as anticholinergic polypharmacy or the absence of intraoperative autonomic measurement.

Finally, this is a narrative review: although the SANRA recommendations have been followed and the evidence graded using the OCEBM 2011 system, the limitations inherent to this design persist—the absence of a formal systematic methodology (PRISMA/PROSPERO), possible selection and citation bias, and lower reproducibility than a systematic review—.

## 9 Future research directions

Clinical validation of the brain–heart–immune framework (BHI-A) will require a research agenda focused on standardising the perioperative acquisition of HRV and baroreflex sensitivity, as well as on the multicentre validation of autonomic adaptability metrics such as DFAα1 and SD2/SD1 for predicting postoperative delirium (POD). Clinical trials are needed to assess whether strategies aimed at enhancing autonomic resilience—including multimodal prehabilitation, HRV biofeedback, transcutaneous auricular vagus nerve stimulation (taVNS), electroencephalographic monitoring, individualised haemodynamic management and nociception-guided analgesia—can reduce the incidence of POD and improve long-term cognitive recovery (49, 50, 55, 64). Likewise, individualised EEG-based anaesthetic strategies should be explored, including the possible influence of anaesthetic-agent choice in patients at risk of burst suppression (58). Wearable devices represent a particularly attractive opportunity to characterise baseline autonomic function and to monitor continuous physiological trajectories outside the hospital (39, 40).

Finally, the integration of autonomic, electroencephalographic, inflammatory, cognitive and frailty data through machine-learning models could facilitate a personalised stratification of POD risk, provided that such models are interpretable and externally validated before clinical application (7, 8, 18, 56).

## 10 Conclusion

The autonomic nervous system emerges as the integrative pathophysiological framework connecting frailty, neuroinflammation, cerebral vulnerability and postoperative delirium, and it accounts for what traditional models centred on isolated organs fail to explain (12, 14). Recent evidence suggests that autonomic adaptability, rather than baseline autonomic state, may constitute a key determinant of perioperative neurobiological resilience: in this respect, the physiological-complexity metrics DFA*α*1 and SD2/SD1 appear to offer predictive capacity superior to that of conventional heart rate variability parameters for stratifying POD risk. Nevertheless, this remains a hypothesis grounded in preliminary and as-yet-unconfirmed evidence, which requires prospective validation (40).

These findings underpin an integrated perioperative strategy, supported by three complementary pillars: autonomic assessment and prehabilitation, to recognise and optimise the patient’s physiological reserve (32, 33); multimodal electroencephalographic monitoring, to individualise anaesthetic administration and avoid the cerebral states associated with neurocognitive vulnerability (58, 69); and nociception surveillance, to guide analgesic titration and contain the physiological burden of surgical stress (55, 62).

Ultimately, we propose that the brain–heart–immune axis offers an integrative pathophysiological framework for understanding perioperative cerebral vulnerability. From this perspective, preoperative autonomic resilience, assessed non-invasively, could constitute the link between frailty, the surgical stress response and the development of postoperative delirium. Its assessment could help to identify the vulnerable brain and to underpin more precise and personalised prehabilitation strategies, shifting the clinical focus from treating established delirium towards its prevention. Nevertheless, this proposal should be understood as a pathophysiological hypothesis with translational potential, whose incorporation into clinical practice requires prospective validation.

## Glossary

3D-CAM: three-dimensional Confusion Assessment Method
4AT: 4 A’s Test (rapid delirium screening instrument)
ABCDEF: bundle of measures for delirium prevention (Assess, Both, Choice, Delirium, Early mobility, Family)
ANI: Analgesia Nociception Index (nociception index derived from HRV)
AUC: area under the ROC curve
BBB: blood–brain barrier
BHI-A: brain–heart–immune axis
BRS: baroreflex sensitivity
BS: burst suppression
CAP: cholinergic anti-inflammatory pathway
CAM: Confusion Assessment Method
CFS: Clinical Frailty Scale
CNAP: continuous non-invasive arterial pressure
DFAα1: alpha-1 exponent of detrended fluctuation analysis
POD: postoperative delirium
DSM-5: Diagnostic and Statistical Manual of Mental Disorders, 5th edition
ECG: electrocardiogram
EEG: electroencephalography
ERAS: Enhanced Recovery after Surgery (multimodal rehabilitation)
ESAIC: European Society of Anaesthesiology and Intensive Care
GFAP: glial fibrillary acidic protein
HF: high frequency (HRV spectral band, 0.15–0.40 Hz)
HFnu: high-frequency power in normalised units
IL-1β/IL-6: interleukin-1 beta/interleukin-6
LF: low frequency (HRV spectral band, 0.04–0.15 Hz)
MoCA: Montreal Cognitive Assessment
NF-kB: Nuclear Factor Kappa-Beta
NIRS: near-infrared spectroscopy
NLRP3: NOD-like receptor protein 3 inflammasome
NOL: Nociception Level Index
PPG: photoplethysmography
RMSSD: Root mean square of successive differences
rSO₂: Regional cerebral oxygen saturation
SD1/SD2: Standard deviations of the Poincaré plot (short and long term)
SDNN: Standard deviation of NN intervals
ANS: Autonomic nervous system
taVNS: Transcutaneous auricular vagus nerve stimulation
IVA: Total intravenous anaesthesia
TNF-α: tumour necrosis factor alpha
HRV: Heart rate variability
α7nAChR: alpha-7 nicotinic acetylcholine receptor
